# Factors Associated with Frailty in Older Adults in Community and Nursing Home Settings: A Systematic Review with a Meta-Analysis

**DOI:** 10.3390/jcm13082382

**Published:** 2024-04-19

**Authors:** Jia Liu, Yuezhi Zhu, Jen Kit Tan, Azera Hasra Ismail, Roszita Ibrahim, Nor Haty Hassan

**Affiliations:** 1 Department of Nursing, Faculty of Medicine, Universiti Kebangsaan Malaysia, Kuala Lumpur 56000, Malaysia; p129290@siswa.ukm.edu.my (J.L.); azera@ppukm.ukm.edu.my (A.H.I.); 2 Department of Biochemistry, Faculty of Medicine, Universiti Kebangsaan Malaysia, Kuala Lumpur 56000, Malaysia; p118017@siswa.ukm.edu.my (Y.Z.); jenkittan@ukm.edu.my (J.K.T.); 3 Department of Public Health Medicine, Faculty of Medicine, Universiti Kebangsaan Malaysia, Kuala Lumpur 56000, Malaysia; roszita@ppukm.ukm.edu.my

**Keywords:** frailty, older adults, associated factors, nursing home, community

## Abstract

**Background:** Frailty is a globally recognized issue. However, there is a lack of evidence exploring factors associated with frailty among older residents in community and nursing-home settings. **Methods:** To explore the prevalence and factors associated with frailty among older adults in community and nursing-home settings, we conducted a systematic search following the PRISMA guidelines across Web of Science, MEDLINE, EMBASE, PubMed, and Cochrane databases up until January 2024, selecting 38 studies which encompassed 150,642 participants. **Results:** Our findings showed higher frailty prevalence in nursing homes compared to communities. Frailty was significantly associated with sociodemographic (living alone, poor self-reported health), physiological (poor sleep, low activity of daily living), behavioral (physical inactivity) and disease (chronic conditions, depression) factors in both community and nursing-home settings. **Conclusions:** There are numerous factors associated with frailty in older adults in nursing-home and community settings. These factors underscore the significance of promptly identifying high-risk individuals and devising appropriate interventions to mitigate frailty among them.

## 1. Introduction

With the advancement of socio-economic factors, there is a continuous expansion in human life expectancy, leading to an increasingly severe challenge posed by an aging population. It is projected that by 2030, the global population aged 60 and above will rise from 1 billion in 2020 to approximately 1.4 billion, accounting for around 17% of the total population [1]. The persistent growth in older adults’ population brings forth diverse health challenges, encompassing physiological aging, prevalent chronic diseases, cognitive decline, malnutrition, physical deterioration, and psychosocial issues. Frailty, increasingly acknowledged as a critical global health challenge among older adults, is defined as a biological syndrome characterized by diminished physiological reserves and heightened vulnerability to external stressors [2,3,4]. Additionally, emerging evidence suggests that among comorbidities for cognitive disorders and frailty, conditions such as sleep apnea should not be overlooked [5]. Sleep apnea, significantly associated with dementia, exemplifies the complex interplay between sleep disorders and the broader health challenges faced by older adults [5]. Therefore, it is evident that frailty is associated with various health issues in older adults.

Up to the present, a total of 67 assessment instruments for frailty screening, grounded in survey indices, clinical data, or their integration, have been formulated. However, a globally standardized assessment tool remains to be established [6,7]. Assessment instruments for frailty are commonly categorized into two distinct groups: the unidimensional, which primarily addresses physical and physiological dimensions, exemplified by the Fried frailty criteria underpinning the Cardiovascular Health Study Index (CHS), or the Physical Frailty Phenotype (PFP); and the multidimensional, encompassing the intricate interplay of physical, psychological, and social dimensions, as seen in tools like the Deficit Accumulation Index (DAI) and the Edmonton Frail Scale (EFS) [6,8,9,10]. Additionally, it is worth noting that not only do these dimensions of frailty reflect the health status of older adults, but physical, psychological, and social frailty have also been found to be predictive of heart failure, underscoring the multifaceted impact of frailty on overall health outcomes [11].

Existing systematic reviews on frailty have primarily focused on prevalence studies, leaving a gap in the analysis of factors influencing frailty among residents in nursing homes and community settings [12,13]. Moreover, frailty is closely associated with a myriad of factors, encompassing age, adverse life events, pain, malnutrition, complications, sleep disorders, and depression, among others [14,15,16,17]. Despite this, earlier research has identified variations in the factors linked to frailty, culminating in inconsistent and at times contentious outcomes [18,19,20,21]. Therefore, this study, through a systematic review and meta-analysis, aims to answer the following questions: What is the prevalence of frailty among older adults in nursing home and community settings? Which factors are significantly associated with the state of frailty among older adults? This endeavor seeks to assist clinical practitioners in implementing early screening and preventive strategies, and to guide healthcare professionals in formulating health prevention policies tailored to the needs of residents in nursing homes and the community.

## 2. Materials and Methods

This study adhered rigorously to the guidelines and methodological principles delineated in the Preferred Reporting Items for Systematic Reviews and Meta-Analyses (PRISMA) statement [22]. Furthermore, the pre-defined review protocol has been registered in the international prospective register of systematic reviews (PROSPERO registration number: CRD42024511189).

### 2.1. Search Strategy

Two researchers independently conducted searches in MEDLINE, EMBASE, PubMed, Web of Science, and the Cochrane Central Register of Controlled Trials from the time of database construction until January 2024. To search these databases, we used the following text words: “elderly”, “aged”, “older adults”, “older people”, “frailty”, “frail”, “debilit* ”, “risk factors”, “associated factors”, “precipitating factors”, “influence factors”, and “contributing factors”, as well as relevant Medical Subject Headings (MeSH) terms. During the search process, this study employed the asterisk (*) as a wildcard to include all variations of the root word ‘debilit’, such as ‘debilitate’ and ‘debilitating’, to ensure the comprehensiveness of our search results. To ensure comprehensive coverage of the literature on frailty among older adults, these databases were selected for their extensive collection of medical and health sciences literature. MEDLINE and PubMed are recognized for their broad coverage of the biomedical literature, including a wide range of subjects pertinent to our research focus. EMBASE offers extensive coverage of drug research and pharmacology, crucial for understanding the medical aspects of frailty, while the Cochrane Central Register of Controlled Trials provides a comprehensive collection of randomized and quasi-randomized controlled trials, essential for reviewing the effectiveness of interventions on frailty. The Web of Science, with its multidisciplinary view, ensures the inclusion of studies from a broad spectrum of scientific fields. This selection strategy aimed at capturing the most relevant and high-quality studies on frailty across different healthcare domains. Detailed information for the search strategy is shown in Supplemental File S1.

### 2.2. Inclusion and Exclusion Criteria

The inclusion criteria for the study encompassed the following (PICO): (1) P: all participants are aged 60 years and above, including those living in nursing homes and community settings; (2) I: research on various factors associated with frailty status; (3) O: studies included must utilize clear, reliable tools or criteria for assessing frailty in the older-adult population and analyze factors associated with frailty; and (4) others: in instances where multiple publications were derived from the same dataset, solely the report most relevant to the study’s focus was included. The comparison (C) aspect of the PICO structure was omitted, as our objective was to examine the factors influencing frailty, rather than to compare effects across different groups or conditions.

The exclusion criteria for the study encompassed the following: (1) studies that did not explicitly report the diagnostic criteria for frailty; (2) conference abstracts, reviews, editorials, case reports, and letters; (3) publications not in English.

### 2.3. Study Selection

Two reviewers independently screened the literature. Initially, duplicates were eliminated using Endnote X9 9.3.3 software. Subsequently, titles and abstracts were assessed for relevance. Finally, full texts of selected articles were thoroughly reviewed to ascertain eligibility. Exclusion reasons for each article in the subsequent phases were documented. Any disagreements were resolved by a third reviewer.

### 2.4. Data Extraction

An independent reviewer (JL) performed data extraction utilizing pre-established forms, and the extracted data were subsequently cross-checked by another reviewer (YZ) for accuracy. Each reviewer independently assessed the inclusion and exclusion criteria for every study, ensuring objectivity. The rationale for exclusion of each study was meticulously recorded in the subsequent phase. All discrepancies encountered were resolved through comprehensive discussions among the reviewers. The information extracted included study design (observational, cohort, cross-sectional, or randomized controlled trials), methodology (sample selection, data collection, and analysis methods), frailty-assessment measures (such as the Fried Frailty Index and the Frailty Index), geographic location, sample demographics (gender and mean age), prevalence of frailty, key findings (risk and protective factors), and conclusions.

### 2.5. Quality Assessment

Two independent researchers systematically appraised the quality of the included studies, employing standardized assessment tools. The Joanna Briggs Institute Critical Appraisal Checklist for Cross-Sectional Studies was utilized [23]. This checklist consists of eight items, each assessed with responses of “not applicable”,“unclear”, “no”, or “yes”. The total count of “yes” responses was recorded for each study. A higher number of “yes” responses indicates higher study quality. Furthermore, the quality of cohort studies was evaluated employing the Newcastle–Ottawa Scale (NOS) [24]. The NOS consists of three categories with eight items in total, with a maximum score of 9. Studies with a NOS score of 5 or less are considered to have a higher risk of bias, those with scores of 5 to 7 have a moderate risk, and those with scores of 7 or more have a low risk. Discrepancies that emerged were reconciled through the mediation of a third researcher.

### 2.6. Statistic Analysis

A meta-analysis was conducted using Revman 5.4.1 software, setting the threshold for statistical significance at *p* < 0.05. For each factor, Odds Ratios (ORs) and 95% Confidence Intervals (CIs) were extracted, followed by the computation of pooled ORs and 95% CIs. Heterogeneity among studies was assessed using chi-square tests and was quantified employing the I^2^ statistic. When *p* ≤ 0.10 and I^2^ ≥ 50% were observed, indicating substantial heterogeneity, a random-effects model was implemented. Otherwise, a fixed-effects model was employed. We assessed the publication bias using funnel plot.

## 3. Results

### 3.1. Search Results

**Figure 1:**
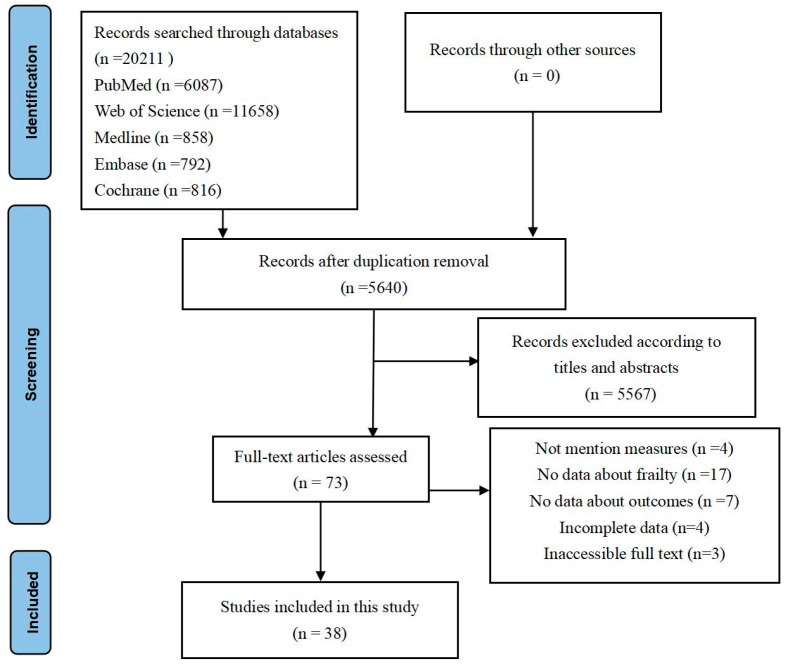
PRISMA diagram for study selection.

During the preliminary literature search, an aggregate of 59,385 studies was retrieved, of which 53,745 were ascertained to be duplicates. Subsequent to the evaluation of titles and abstracts in accordance with the inclusion criteria, 73 studies were ultimately selected for further analysis. Following an exhaustive assessment of the full texts, a total of 38 articles were adjudged to be appropriate for inclusion in the meta-analysis (Figure 1).

### 3.2. Characteristics of Included Studies

The studies encompassed the participation of 150,642 individuals, including 32,936 males and 43,455 females. Of these, 6204 individuals were residents of nursing homes (as reported in 8 studies [25,26,27,28,29,30,31,32]), whereas 144,438 individuals were from community settings (as detailed in 30 studies [33,34,35,36,37,38,39,40,41,42,43,44,45,46,47,48,49,50,51,52,53,54,55,56,57,58,59,60,61,62]). It is noteworthy that three studies [31,47,62] did not make a distinction between male and female participants. Of the entire corpus of studies, 20 [25,26,27,30,32,33,34,37,41,45,47,48,49,50,53,57,58,61,62] adopted a cross-sectional design, whereas the remaining 18 [29,31,35,36,38,39,40,42,43,44,46,51,52,54,55,56,59,60] constituted cohort studies. The detailed characteristics of the 38 studies included are systematically presented in Supplemental File S2 (Table S1).

Within this collection of studies, 23 were undertaken in various Asian countries, while 15 originated from other nations, including 8 from Europe [29,37,39,46,48,51,57,58] and 7 from the Americas [28,30,44,45,49,56,62].

With regard to the assessment instruments utilized, the Physical Frailty Phenotype (PFP)/CHS Frailty emerged as the predominant standard employed across both nursing home and community-dwelling contexts. More specifically, the PFP was applied in seven nursing home-based studies and thirteen studies within community settings. Furthermore, a range of tools including the FRAIL scale, Clinical Frailty Scale, Kihon Checklist (KCL), Frailty Staging System (FSS), Frailty Index (FI)/Deficit Accumulation Index (DAI), Groningen Frailty Indicator (GFI), Edmonton Frailty Scale (EFS), and Tilburg Frailty Indicator (TFI) were employed in the community-dwelling settings of this study. The estimated total uses and features of frailty instruments in the literature are shown in Supplemental File S2 (Table S2).

### 3.3. Quality Assessment of the Included Studies

The results of the quality evaluation for the 13 cross-sectional studies and 25 cohort studies are shown in Supplemental File S3. The quality evaluation of the 13 cross-sectional studies was conducted using the Joanna Briggs Institute Critical Appraisal Checklist. Within these studies, two satisfied six items of the checklist, four fulfilled the criteria for seven items, and the remaining seven adhered to all eight items. Regarding the 25 cohort studies, their quality was assessed employing the Newcastle–Ottawa Scale. One study attained a score of 8, whereas the other 24 studies each scored 9, denoting high-quality research. The results indicate a high quality of the studies.

### 3.4. Publication Bias

Statistical tests for the asymmetry of scatter plots and reporting bias were not performed due to the uncertainty of bias risk of most studies, high statistical heterogeneity (>90%), and the limited number of studies (<10). Further details are shown in Supplemental File S4.

### 3.5. Associated Factors of Frailty in Older Adults in Nursing Home and Community Settings

The studies encompassing older adults in nursing homes and community settings revealed that the prevalence of frailty in nursing homes fluctuated between 10% and 80.1%, whereas in community settings, it varied from 3.8% to 70.6%. The factors associated with frailty were systematically classified into social demographic (age, gender, household size, educational level, self-reported health status, and marital status), physiological (level of activity of daily living (ADL), body mass index (BMI), nutritional status, medication status, and sleep quality status), behavioral (level of physical activity), and disease-related (chronic conditions, cognitive impairment, depression, and hypertension) factors. Among these, age, physical inactivity, and cognitive impairment were the associated factors with frailty in nursing homes. Age, living alone, low education, unmarried/divorced/widowed, low BMI, polypharmacy, malnutrition, low ADL, poor sleep, physical inactivity, chronic conditions, cognitive impairment, and depression were the associated factors in community settings. However, in both nursing home and community settings, there was no significance in low education and unmarried/divorced/widowed, but there was for female, poor self-reported health, and hypertension. Details are summarized in Table 1, and the forest plots are shown in Supplemental File S4. 

## 4. Discussion

The study indicates that in terms of frailty assessment tools, at the unidimensional analysis level, researchers show a marked preference for the Physical Frailty Phenotype (PFP) proposed by Fried to define frailty among nursing-home residents. Additionally, it has been specifically noted that among 67 frailty assessment tools, only the PFP and the Deficit Accumulation Index (DAI) are suitable for assessing frailty risk factors [6]. The PFP is primarily used in epidemiological, clinical, and intervention studies, while the DAI is mainly employed in large-scale database research, in addition to epidemiological and clinical studies [7]. Regarding the Frail scale, its frailty assessment is predominantly conducted within community populations; the Clinical Frailty Scale and the EFS assessments are primarily conducted in hospital or clinical settings, which diverges from the findings of this study. In contrast, the KCL, FSS, GFI, and TFI are applicable in both community and clinical settings [6,7,10,64]. However, it is noteworthy that in the nursing home environment, the studies included in this research did not use the FRAIL-NH tool, which is specifically tailored for assessing frailty in nursing-home populations [65,66]. 

Furthermore, this study found that the prevalence of frailty in nursing-home settings is relatively high, showing a significant difference compared to community environments, a finding that aligns with previous research [64]. This disparity could be attributed to, first, the currently limited number of studies focused on nursing homes; second, the inclusion of studies using different frailty assessment tools, leading to heterogeneity in the estimates of frailty prevalence [67,68]; and third, the structured environment of nursing homes may facilitate professional caregivers in more easily identified and documented cases of frailty.

Based on this study, when assessing frailty, it is important to recognize that frailty is not limited to physical muscle-level weakness, but also includes a general vulnerability of the individual to various external stressors. Screening for frailty should consider multidimensional tools that assess physical, psychological, and social factors, as unidimensional tools may underestimate the prevalence. Additionally, conducting longitudinal studies to evaluate the predictive effectiveness of various frailty assessment tools in different settings and populations allows for a more in-depth understanding of the strengths and limitations of these tools, particularly in terms of their ability to predict frailty risk across diverse environments and demographic groups. 

### 4.1. Socio-Demographic Factors Associated with Frailty in Nursing Homes and Community Settings

The outcomes of this research clearly indicate that age constitutes a significant socio-demographic factor, impacting the manifestation of frailty among residents in both nursing homes and community settings. With advancing age, individuals experience a progressive decline in muscle mass and functionality, accompanied by reduced bone density, factors that collectively escalate the risk of frailty [69]. Older adults more frequently encounter the prevalence of chronic diseases and the coexistence of multiple health conditions, leading to the impairment of various bodily systems, thereby increasing individuals’ susceptibility to frailty [70]. Social determinants, such as social isolation and loneliness, potentially exacerbated by aging, can adversely affect mental health, subsequently elevating the risk of frailty [71]. A study focusing on frailty among community-dwelling older people also reveals that frailty prevalence intensifies with age, with notably higher prevalence rates observed in older age cohorts compared to their younger counterparts [69,70]. The prevalence rates of frailty are 4% among those aged 65–69, escalating to 7% for ages 70–74, 9% for 75–79, 16% for 80–84, and 26% for individuals aged 85 and above [14,66,72,73]. 

Residing alone, possessing a lower level of education, and being unmarried, divorced, or widowed are identified as independent risk factors contributing to frailty among residents living in community settings. Elderly individuals who reside alone are particularly susceptible to frailty. The absence of companionship and social interaction can precipitate physical frailty, a situation further exacerbated by unhealthy lifestyle habits, mental-health challenges, and the prevalence of chronic diseases. Evidence indicates that although not all elderly individuals living alone exhibit physical frailty, many may encounter heightened psychological distress, loneliness, and a lack of social support, rendering them more vulnerable to various environmental challenges, which in turn amplifies their overall susceptibility to frailty [74]. Nonetheless, certain studies have suggested that living alone might serve as a protective factor against the onset of frailty, although the exact mechanisms behind this protection are not entirely clear. It is hypothesized that elderly individuals residing alone might exhibit greater physical and psychological independence in their daily activities, thereby being less prone to exhibiting symptoms of frailty or facing a reduced risk of physical frailty [75]. 

Furthermore, it has been observed that community-dwelling older adults who have attained a university education or higher typically exhibit a reduced degree of frailty. This observation is in alignment with the findings of prior research studies [34,71]. An individual’s level of education is intricately linked to their health literacy, coping mechanisms, and the propensity to adhere to healthy lifestyle practices. Elderly individuals with higher education levels are more likely to adopt healthier lifestyles, possess enhanced health literacy, and exhibit stronger coping skills, thereby significantly reducing the risk of frailty and enhancing overall well-being and health status.

The status of being unmarried, divorced, or widowed also stands as an independent risk factor for frailty among elderly individuals residing in communities. This observation aligns with the findings of related studies conducted in Brazil and Mexico [75,76]. Considering that marital status often forms a critical component of the social support structure for older adults, the absence of such social support relationships could heighten the risk of psychosocial issues. Consequently, this state of compromised social–psychological health might adversely affect the overall health of elderly individuals at a societal level, thereby increasing the likelihood of frailty.

Intriguingly, our study discovered that although gender and poor self-reported health were not significantly associated with frailty in isolated analyses of both community and nursing-home environments; a significant pattern emerged when data from both environments were amalgamated. The comprehensive analysis elucidated that gender and self-reported poor health exert a statistically significant influence on frailty, highlighting the critical importance of considering the combined effects of these variables in diverse living conditions. The observation that women are more susceptible to frailty compared to men is consistent with the conclusions of preceding studies [77,78,79]. This phenomenon could be attributed to a survival effect, evidenced by higher mortality rates in men across all major diseases, including but not limited to cardiovascular diseases, malignant tumors, chronic obstructive pulmonary disease, pneumonia, and accidental injuries [80,81]. Furthermore, older women are more likely to accumulate abdominal fat, which may contribute to elevated levels of inflammatory factors (such as IL-6 and CRP), which are associated with the development of frailty [41]. A cross-sectional study in Spain involving individuals over 65 years of age revealed gender differences in the relationship between testosterone levels and frailty, manifesting as a negative correlation in men and a “U”-shaped relationship in women [82]. Contrarily, certain studies have observed that frailty, as assessed using the FP instrument, is more prevalent in men [83]. Additionally, two separate studies conducted in Canada and Singapore reported no significant correlation between gender and the prevalence of frailty [84,85]. Furthermore, an individual’s subjective assessment of their health status holds a significant predictive value in determining frailty. Research has shown that when individuals self-assess their health as poor, this evaluation is frequently associated with a spectrum of physiological, psychological, and social issues pertinent to frailty [86]. This association may be partly attributed to an individual’s heightened sensitivity to underlying health issues and the precision of their self-perception. In the process of assessing their own health, individuals often take into account various sensations and experiences such as fatigue, diminished capacity to perform activities, and social isolation, all of which are intricately linked to frailty.

Based on the findings, a multifaceted approach is recommended to mitigate these impacts and enhance the overall well-being of this population group. Initially, targeted interventions such as strength training and nutritional support programs should be implemented to address the decrease in bone density and muscle mass associated with advancing age. Additionally, enhancing social connections and providing lifelong learning opportunities can effectively prevent frailty risks associated with living alone and lower educational levels. For those who are unmarried, divorced, or widowed, strengthening social support systems and offering emotional and social assistance are crucial in navigating psychosocial challenges. Considering the differences in frailty risk between genders, health-promotion strategies tailored to the unique needs of men and women should be developed, focusing on their specific health requirements. Encouraging the self-assessment of health among older adults can foster awareness of their health status, enabling the timely identification of potential frailty risks. Finally, interdisciplinary collaboration among healthcare, social work, and community organizations is encouraged to create a comprehensive support environment for older adults to prevent and manage frailty.

### 4.2. Physiological Factors Associated with Frailty in Nursing Homes and Community Settings

This study underscores that those factors such as low BMI, malnutrition, polypharmacy, poor sleep quality, and low ADL are significant risk factors for frailty in community-dwelling individuals. Low BMI and malnutrition are identified as risk factors for frailty, predominantly because a low BMI is frequently indicative of inadequate nutrition, which can lead to decreased muscle mass and bone density, consequently elevating the risk of frailty [1,87]. Some studies have highlighted that malnutrition can precipitate weight loss and physical deterioration, particularly when energy intake is ≤21 kcal/kg, a condition that has a significant association with frailty [87,88]. However, studies have demonstrated that both excessively high and low BMI levels are associated with an increased risk of frailty, in comparison to individuals with a normal BMI range [89]. In a study undertaken in Japan, the minimal prevalence of frailty among elderly individuals was observed within a BMI range of 21.4–25.7 kg/m^2^, indicating a pronounced “U”-shaped association between BMI and frailty prevalence [90]. This suggests that rates of frailty are lower within an intermediate BMI range, but tend to rise at both higher and lower extremes of BMI. The interplay between frailty, weight fluctuations, and malnutrition is governed by intricate biological mechanisms. Although frailty is characterized as a catabolic state, and weight loss might be one of its manifestations, it is not an obligatory condition [19]. Conversely, overweight and obesity can directly lead to diminished mobility and impaired physical performance, contributing to the development of frailty [91]. Furthermore, individuals with obesity may undergo a decline in anabolic hormone levels, which is associated with diminished muscle strength and could potentially lead to muscle deterioration [91,92]. Lastly, obesity frequently coexists with chronic conditions such as cardiovascular diseases, insulin resistance, and diabetes, each of which can further exacerbate the risk of developing frailty [3,93,94]. These insights collectively unveil the intricate and multifaceted relationship between frailty, body weight, and nutritional status, necessitating an in-depth and comprehensive consideration of a multitude of factors to gain a more profound understanding of the development of frailty.

Polypharmacy has been recognized as an independent risk factor for frailty, in alignment with current scientific evidence [95]. In older adults, polypharmacy not only mirrors the underlying health conditions but also acts as a marker of heightened risk for drug-related toxicity. Although the concept of polypharmacy is widely utilized, it suffers from a lack of a unified and explicit definition [96]. The disparate definitions of polypharmacy in existing research (which range from the use of more than three to six or more medications) can lead to confusion and challenges in comparing study outcomes and formulating general conclusions. In this study, polypharmacy was characterized as the concurrent use of three or more medications. This relatively lower threshold may elucidate the challenges encountered in establishing a significant correlation between multiple-medication usage and frailty. For instance, Trevisan et al. [97] observed no correlation between the use of three or more medications and the transition to pre-frailty or frailty in their multivariate analysis, possibly due to the low threshold of three medications. In a research study focusing on men over 70 residing in Australian communities, Gnjidic et al. [98] identified a cutoff of 6.5 medications as the optimal threshold for identifying frailty. Similarly, a study involving French men and women over the age of 65 reported a threshold of at least six medications [99]. Subsequent investigations into the nexus between polypharmacy and frailty could benefit from adopting this specific threshold as a standard for defining polypharmacy. Alternatively, employing the average number of medications used, as opposed to a strict cutoff, may yield more nuanced and comprehensive insights into this complex relationship [100].

This study elucidated a notable association between poor sleep quality, characterized by difficulty in maintaining and initiating sleep, and the incidence of frailty. Numerous vital restorative processes occur in various systems of the human body during sleep, and diminished sleep quality may hasten the deterioration of functional capacities and physiological reserves [101]. Additionally, sleep disorders are frequently associated with psychological challenges and a consequent decline in quality of life [102]. Concurrently, among older people, a range of factors, notably medication use, contribute to an augmented susceptibility to sleep disorders, which subsequently amplifies the risk of developing frailty [103].

In addition, low ADL is recognized as a risk factor for the onset of frailty, indicating that functional impairments in individuals can precipitate frailty [104,105]. Firstly, low ADL frequently signifies a decline in physiological functions, encompassing reduced muscle strength, balance, coordination, and mobility, all of which are characteristic hallmarks of frailty. Secondly, low ADL can result in diminished mobility, impacting an individual’s ability to perform essential tasks such as walking, climbing stairs, and carrying objects, thereby directly influencing the progression of frailty. Thirdly, low ADL is frequently associated with impaired balance and gait stability, consequently elevating the likelihood of falls. Falls constitute a significant risk factor for grave outcomes such as fractures and are intimately linked with the progression of frailty. Furthermore, the presence of chronic diseases and inflammation is often concomitant with low ADL, hastening the advancement of frailty. Lastly, a deterioration in ADL can interact synergistically with psychological health concerns. Mental health issues can impede active engagement in daily activities, thereby further aggravating the state of frailty.

Intriguingly, upon separate analysis of nursing home environments, the correlation between the aforementioned factors and frailty appears to be non-significant. This discrepancy in environments may be indicative of the unique attributes distinguishing community dwellings from nursing-home settings. Within community environments, there is typically an augmented emphasis on individual autonomy and independence. Consequently, determinants of frailty in these settings may be more intricately linked to an individual’s physical status, capabilities in daily living, and lifestyle choices. Conversely, in nursing homes, the provision of more professional medical and nursing care might attenuate the significance of traditionally frailty-associated factors in this context.

Based on the findings, a comprehensive strategy is recommended to mitigate the impact of physiological factors on the older adults. Firstly, nutritional interventions aimed at maintaining an optimal BMI range should be implemented to address the risks of frailty associated with malnutrition or obesity, including regular assessments of nutritional status and dietary recommendations for older adults. Secondly, routine medication reviews are advocated for to minimize the risks associated with polypharmacy, especially among older adults, by adjusting medication regimes to reduce unnecessary drug use. The screening and management of sleep disorders, along with the promotion of good sleep hygiene practices, are recommended to improve sleep quality and decrease the risk of frailty. Thirdly, for individuals with low ADL, physical therapy and strength training are suggested to enhance their physical function and independence. Lastly, tailored care plans should be developed, considering the specific needs of nursing home and community settings, focusing on maintaining and enhancing residents’ physical and cognitive functions to effectively address frailty.

### 4.3. Behavioral Factors Associated with Frailty in Nursing Homes and Community Settings

Prior studies have underscored the significant association between frailty and sarcopenia, highlighting its importance in geriatric research. Consequently, the effective prevention and treatment of sarcopenia are of paramount importance for the well-being of the frail elderly population [106]. Physical exercise has been empirically validated as an efficacious approach. This intervention can markedly enhance the physical functionality and life quality of frail older adults, diminish the incidence of falls, and assure safety and adherence in geriatric physical activities [106,107]. Tai Chi, holistic functional training, and moderate-intensity exercises have been demonstrated to yield positive outcomes in enhancing the physical agility, equilibrium, and reactive capabilities of older individuals [85,108,109]. 

Our study findings underscore that physical inactivity among elderly residents in community and nursing home settings independently contributes to the risk of frailty. The existing literature suggests that regular physical activity is intimately associated with enhanced physiological capacity in individuals. This is attributed to the adaptive modifications within the physiological systems, notably the neuromuscular system’s coordination of movement, the cardiopulmonary system’s enhanced distribution of oxygen and nutrients, and an improved regulation of glucose and fatty acid metabolism [86]. However, a survey conducted in England revealed a gradual decrease in sports participation and dwindling interest in physical exercise with increasing age. Merely half of the adult population and about one-fourth of individuals over 65 achieve the minimum recommended levels of physical activity [110]. This lack of activity is a significant cause of deteriorated physiological health and diseases in older adults, with consequences akin to those of smoking, excessive alcohol consumption, and obesity [106,107]. Consequently, with advancing age, there is a tendency for reduced participation in physical activities, potentially impacting overall health negatively.

Based on the findings, the recommendations of this study are as follows: firstly, it is crucial to integrate physical exercises into the daily routines of older adults, such as Tai Chi, holistic functional training, and moderate-intensity exercises, as these activities have been proven to improve physical agility, balance, and reactive capabilities in older adults, while also reducing the incidence of falls and ensuring safety and adherence in sports activities. Additionally, given that physical inactivity is a significant independent risk factor for frailty, emphasizing the importance of regular physical activity to enhance physiological capacity and prevent diseases associated with sedentary lifestyles is essential. To this end, we propose the development and implementation of strategies to increase the levels of physical activity among older adults, especially addressing the decline in sports participation and interest in physical exercise with advancing age. Community and healthcare providers should develop accessible, age-appropriate physical activity programs to combat the effects of aging and improve the overall health and quality of life for older people.

### 4.4. Disease-Related Factors Associated with Frailty in Nursing Homes and Community Settings

Our research findings indicate a positive correlation between cognitive impairment and frailty among elderly residents in nursing homes and community settings. However, the association between frailty and cognitive impairment continues to exhibit a spectrum of discrepancies and controversies [111]. Brigola et al. suggest that individuals with frailty exhibit lower scores on the Mini-Mental State Examination (MMSE) compared to robust individuals, predominantly demonstrating memory impairments [112]. However, the cross-sectional study conducted by Lorenzo-López et al. found no significant differences in MMSE scores between frail and robust individuals, yet noted lower Montreal Cognitive Assessment (MoCA) scores in the frail group, especially in immediate and delayed memory subtests [113]. This finding contradicts previous studies that propose non-memory domains are typically the first to be compromised in the prefrail state. A study suggests that executive function and attention are associated with frailty, whereas memory does not exhibit this relationship [114]. Consequently, while there is agreement on the correlation between global cognitive function and frailty, considerable debate persists regarding the link between specific cognitive domains and frailty. These variances can be attributed to the diversity in research design, population characteristics, and the methodologies employed in measurement tools. 

Additional significant factors associated with frailty in community-dwelling older people encompass chronic conditions and depression. Older adults harboring multiple chronic diseases are at an increased risk of entering a deleterious cycle owing to the potential interactions among these ailments [115], thereby hastening the decline in their overall health status [116]. Additionally, a longitudinal study involving 7439 individuals aged over 65 revealed that frailty serves as a robust predictor of disability and elevated comorbidity rates [117]. Older adults suffering from depression may exhibit diminished physical activity, reduced mobility, and decreased nutritional intake, consequentially leading to the degradation of muscle function and strength, thereby amplifying the probability of frailty. Additionally, there is mounting evidence indicating a bidirectional causal relationship between frailty and depression. On one hand, frailty may precipitate adverse outcomes including falls, increased medical expenditures, and reduced social interaction, potentially triggering anxiety and culminating in depression [118]. On the other hand, depression can result in poor nutritional status, sleep disturbances, and emotional dysregulation, each of which can significantly impact physical health and contribute to the development of frailty [119]. Moreover, accumulating evidence supports the notion that the bidirectional causality between frailty and depression may be partially elucidated through shared risk factors and pathophysiological pathways, including chronic inflammation, oxidative stress, mitochondrial dysfunction, and the dysregulation of the hypothalamic–pituitary–adrenal axis [120,121]. Another study indicates that depression is the most significant factor contributing to frailty in older adults, due to its comprehensive impact on physiological, behavioral, nutritional, and cognitive aspects [122,123,124]. Physiologically, depression directly leads to reduced muscle mass and function through mechanisms such as chronic inflammation, hormonal imbalances, and decreased immune system functionality. Behaviorally, individuals with depression experience a lack of energy, diminished interest, and reduced activity levels, leading to decreased physical activity and social engagement. Additionally, depression can adversely affect appetite and eating habits, leading to malnutrition, a key risk factor for frailty. Cognitively, depression is associated with a decline in cognitive function, potentially resulting in diminished daily living abilities. Furthermore, elderly individuals with depression often have comorbid chronic diseases, which, combined with the psychological stress of depression, accelerate the development of frailty.

Interestingly, our analysis revealed that when synthesizing combined data from both nursing homes and community settings, hypertension emerged as a notable influencing factor. This observation may suggest that environmental factors significantly influence the relationship between hypertension and frailty. This environmental dependency may encompass disparities in factors such as lifestyle, medical management, social interaction, and other elements between community and nursing-home settings. However, current research on the relationship between frailty and hypertension remains inconclusive: while some longitudinal studies have assessed the impact of hypertension on the development of frailty, their findings are contradictory. Additionally, to date, no studies have investigated whether hypertension could serve as a predictive factor for the onset of frailty. Research indicates that variability in population characteristics, environments, and the adjustment for confounders in different studies can affect outcomes. Conditions such as hypertension, sleep apnea, and lipid disorders can lead to the occurrence of frailty in older adults, yet these conditions may also act as confounding variables, mutually influencing each other and thus altering the results [125]. Finally, meta-analyses of cross-sectional studies have failed to identify a significant association between these two conditions.

Based on the findings, this study emphasizes the importance of monitoring simple electrocardiogram parameters in frail older adults with cognitive impairments. A previous study has substantiated that this approach may assist in preventing or decelerating the progression of cognitive disorders, facilitating the selection of patients for neuropsychological testing or brain-imaging screening, and identifying those who may benefit from pharmacological treatment, particularly those diagnosed with hypertension [126]. Elderly individuals with frailty and chronic diseases should focus on the management of chronic conditions to ensure their overall health and reduce the risk of frailty. Additionally, it is imperative to implement early screening and intervention for depression in frail older adults and establish psychological counseling centers in nursing homes and communities to prevent a downward spiral in health status. 

### 4.5. Strengths and Limitations of the Review

Firstly, this study represents an inaugural systematic review and meta-analysis concentrating on the determinants of frailty among older adults residing in nursing-home and community settings. Secondly, a thorough literature search was performed, encompassing a meticulous screening process of research papers as well as pertinent literature sources. This exhaustive approach aimed to minimize the risk of overlooking any relevant studies. Thirdly, the majority of the studies incorporated into the analysis were characterized by their high quality and produced dependable findings. 

However, this study encompasses specific limitations. Firstly, there is a notable lack of research focusing on frailty within nursing-home environments, and cross-sectional studies dominate the field, limiting the assessment of temporal relationships between factors and the onset of frailty. Secondly, in some studies, the small sample size limits the ability to detect significant associations. Thirdly, there is a risk of publication bias, where studies with significant findings are more likely to be published. Furthermore, although this study selected MEDLINE, EMBASE, PubMed, Web of Science, and the Cochrane Central Register of Controlled Trials to ensure the breadth and depth of the literature search, relying on these databases for the literature review also entails certain limitations in coverage scope; finally, this study’s inclusion of only English-language articles may partially affect the presentation and interpretation of the results.

Therefore, to effectively manage and prevent frailty among older adults, it is necessary to implement personalized, multifactorial interventions in nursing homes and community settings, targeting modifiable risk factors such as nutrition, polypharmacy, and physical activity. Additionally, promoting healthy aging through education, social programs, and chronic disease management, along with developing targeted intervention measures and researching new biomarkers, is crucial for the early identification and prevention of frailty risk. Lastly, it is essential to assess the cost-effectiveness of community-based frailty-prevention programs. A study has shown that frail older adults account for the highest healthcare costs in developed countries [127]. This highlights the importance of evaluating the economic aspects of frailty prevention programs to ensure the sustainability and scalability of such interventions in reducing healthcare costs and improving the quality of life for older adults.

## 5. Conclusions

There are numerous factors contributing to frailty, and a portion of them are modifiable, such as living alone, low educational attainment, low BMI, low ADL, malnutrition, poor sleep quality, and physical inactivity. Additionally, varying frailty-assessment tools not only result in differences in prevalence rates but also lead to disparities in identified influencing factors, such as gender. It is recommended that future frailty assessments in nursing homes utilize Frail-NH to enhance the accuracy of frailty evaluations. Finally, there are discernible differences in the factors influencing frailty in community and nursing-home settings. Recognizing and distinguishing the intertwined factors contributing to frailty in these distinct settings is crucial for accurately implementing targeted intervention measures.

## Figures and Tables

**Table 1:** Pooled ORs and 95% confidence interval for associated factors of frailty in older adults in nursing-home and community-dwelling settings.

Associated Factors		Setting	Number of Studies	Heterogeneity		OR (95% CI)
				I^2^	*p*	
Sociodemographic factors	Age	NH	6	97	0.002	1.83 (1.26–2.66)
Behavior factors	Physical inactivity	NH	4	6	<0.001	2.27 (1.85–2.80)
Disease factors	Cognitive impairment	NH	3	87	0.26	1.11 (0.92–1.34)
Sociodemographic factors	Age	Community	14	97	0.17	1.09 (0.97–1.22)
	Living alone	Community	5	75	0.01	1.68 (1.13–2.51)
	Low education	Community	9	83	0.23	0.89 (0.74–1.08)
	Unmarried/divorced/ Widowed	Community	4	47	<0.001	2.26 (1.65–3.09)
Physiological factors	Low BMI	Community	3	95	0.14	2.14 (0.77–5.93)
	Polypharmacy	Community	9	77	<0.001	1.48 (1.27–1.72)
	Malnutrition	Community	4	99	0.87	1.16 (0.20–6.65)
	Low ADL	Community	8	96	<0.001	1.71 (1.25–2.34)
	Poor sleep	Community	6	88	<0.001	2.40 (1.51–3.83)
Behavioral factors	Physical inactivity	Community	10	95	0.01	1.58 (1.11–2.25)
Disease factors	Chronic conditions	Community	9	93	<0.001	1.94 (1.40–2.70)
	Cognitive impairment	Community	4	98	0.11	1.81 (0.87–3.75)
	Depression	Community	9	94	<0.001	2.02 (1.45–2.82)
Sociodemographic factors	Age	NH and Community	20	100	<0.001	1.43 (1.16–1.75)
	Female	NH and Community	4	91	0.98	0.98 (0.22–4.40)
	Living alone	NH and Community	6	84	0.009	2.11 (1.21–3.70)
	Poor self-reported health	NH and Community	3	50	<0.001	2.04 (1.50–2.76)
Physiological factors	Low ADL	NH and Community	10	95	0.003	1.65 (1.19–2.30)
	Malnutrition	NH and Community	5	99	0.66	1.40 (0.31–6.37)
	Polypharmacy	NH and Community	10	98	<0.001	1.63 (1.25–2.13)
	Poor sleep	NH and Community	7	85	<0.001	2.37 (1.58–3.56)
Behavioral factors	Physical inactivity	NH and Community	14	94	0.003	1.57 (1.17–2.10)
Disease factors	Chronic conditions	NH and Community	10	93	<0.001	2.14 (1.54–2.99)
	Cognitive impairment	NH and Community	7	96	0.003	1.33 (1.10–1.61)
	Depression	NH and Community	11	92	<0.001	1.99 (1.48–2.67)
	Hypertension	NH and Community	3	94	0.67	0.65 (0.09–4.51)

Abbreviations: BMI: Body Mass Index; CI: Confidence Interval; I^2^: I-squared statistic; NH: nursing home; OR: Odds Ratio; ADL: activity of daily living. Note: Malnutrition: a condition where the body cannot meet its health requirements due to insufficient or imbalanced dietary intake. Malnutrition is often associated with poor nutritional status [63].

## Data Availability

The data presented in this study are available in Supplementary File S2.

## Supplementary Materials

The following supporting information can be downloaded at: https://www.mdpi.com/article/10.3390/jcm13082382/s1, Figure S1: Meta-analysis of sociodemographic factors of frailty; Figure S2: Meta-analysis of physiological factors of frailty; Figure S3: Meta-analysis of behavior factors of frailty; Figure S4: Meta-analysis of disease factors of frailty; Figure S5: Risk of bias graph of the included cross-sectional studies; Figure S6: Risk of bias summary of the included cross-sectional studies; Figure S7: Risk of bias graph of the included cohort studies; Figure S8: Risk of bias summary of the included cohort studies; Table S1: Characteristics of studies included in this meta-analysis; Table S2: The estimated total uses and features of frailty instruments in the literature.
