# Supplementary material for: Factors Associated with Frailty in Older Adults in Community and Nursing Home Settings: A Systematic Review with a Meta-Analysis
Source: J Clin Med. 2024 Apr 19;13(8):2382. doi: 10.3390/jcm13082382 (PMC11050860; doi:10.3390/jcm13082382)
Supplement: Supplementary file 1 [file jcm-13-02382-s001.zip › Supplemental File S1 search strategy for each database.pdf]

No	Databases	Search trems
1	PubMed	#1.Search aged[MeSH Terms]<br>#2.Search (aged [Title/Abstract] OR older People [Title/Abstract] OR elderly [Title/Abstract] OR older adults [Title/Abstract])<br>#3.Search(#1 OR #2)<br>#4.Search frailty[MeSH Terms]<br>#5.Search (frailty [Title/Abstract] OR frail [Title/Abstract] OR debilit* [Title/Abstract])<br>#6.Search(#4 OR #5)<br>#7.Search risk factors[MeSH Terms]<br>#8.Search (risk factors [Title/Abstract] OR associated factors [Title/Abstract] OR influence factors [Title/Abstract] OR precipitating factors [Title/Abstract] OR contributing factors [Title/Abstract])<br>#9.Search (#7 OR #8)<br>#10.Search (#3 AND #6 AND #9)
2	MEDLINE	#1.Search aged[MH]<br>#2.Search (aged [AB] OR older people [AB] OR elderly [AB] OR older adults [AB])<br>#3.Search(#1 OR #2)<br>#4.Search frailty[MH]<br>#5.Search (frailty [AB] OR frail [AB] OR debilit* [AB])<br>#6.Search(#4 OR #5)<br>#7.Search risk factors[MH]<br>#8.Search (risk factors [AB] OR associated factors [AB] OR influence factors [AB] OR precipitating factors [AB] OR contributing factors [AB])<br>#9.Search (#7 OR #8)<br>#10.Search (#3 AND #6 AND #9)
3	EMBASE	#1.Search ‘aged’/exp<br>#2.Search (‘aged’:ti.ab.kw OR ‘older people’:ti.ab.kw OR ‘elderly’: ti.ab.kw OR ‘older adults’: ti.ab.kw)<br>#3.Search(#1 OR #2)<br>#4.Search ‘frailty’/exp<br>#5.Search (‘frailty’:ti.ab.kw OR ‘Frail’:ti.ab.kw OR ‘debilit*’:ti.ab.kw)<br>#6.Search(#4 OR #5)<br>#7.Search ‘risk factors’/exp<br>#8.Search (‘risk factors’:ti.ab.kw OR ‘associated factors’:ti.ab.kw OR ‘influence factors’:ti.ab.kw OR ‘precipitating factors’:ti.ab.kw OR ‘contributing factors’:ti.ab.kw)<br>#9.Search (#7 OR #8)<br>#10.Search (#3 AND #6 AND #9)
4	Web of Science	#1.Search (aged[Topic] OR older people[Topic] OR elderly[Topic] OR older adults[Topic])<br>#2.Search (Frailty [Topic] OR Frail [Topic] OR debilit* [Topic])

		#3.Search (risk factors[Topic] OR associated factors[Topic] OR influence factors[Topic] OR precipitating factors[Topic] OR contributing factors [Topic])<br>#4.Search (#1AND #2 AND #3)
5	Cochrane	#1.Search aged[MeSH terms]<br>#2.Search (aged [Title Abstract Keyword] OR older people [Title Abstract Keyword] OR elderly [Title Abstract Keyword] OR older adults [Title Abstract Keyword])<br>#3.Search(#1 OR #2)<br>#4.Search frailty[MeSH terms]<br>#5.Search (frailty [Title Abstract Keyword] OR frail [Title Abstract Keyword] OR debilit* [Title Abstract Keyword])<br>#6.Search(#4 OR #5)<br>#7.Search risk factors[MeSH terms]<br>#8.Search (risk factors [Title Abstract Keyword] OR associated factors [Title Abstract Keyword] OR influence factors [Title Abstract Keyword] OR precipitating factors [Title Abstract Keyword] OR contributing factors [Title Abstract Keyword])<br>#9.Search (#7 OR #8)<br>#10.Search (#3 AND #6 AND #9)
