# Supplementary material for: Factors Associated with Frailty in Older Adults in Community and Nursing Home Settings: A Systematic Review with a Meta-Analysis
Source: J Clin Med. 2024 Apr 19;13(8):2382. doi: 10.3390/jcm13082382 (PMC11050860; doi:10.3390/jcm13082382)
Supplement: Supplementary file 1 [file jcm-13-02382-s001.zip › Supplemental File S2 Tables.pdf]

Table S1. Characteristics of studies included in this meta-analysis

First author(year)	Setting	Region	Study design	Total (N)	Male (N)	Female (N)	Mean age	Age	Measure	Prevalence (N%)
Weiwei Liu(2020)	NH	China	cross-sectional	2630	889	1741	80.8±8.9	≥60	PFP	55.6
Meng Zhao MS(2019)	NH	China	cross-sectional	370	150	220	77.6±8.7	≥60	PFP	29.2
Resshaya Roobini Murukesu (2019)	NH	Malaysia	cross-sectional	302	208	94	68.9±7.2	≥60	PFP	40.7
Mariana Marinho Davino de Medeiros (2020)	NH	Brazil	cross-sectional	344	138	206	77.7±9.1	≥60	PFP	58.1
Marisa de la Rica-Escuín (2014)	NH	Spain	Cohort study	287	100	187	84.2±6.8	≥65	PFP	69.3
Fabienne Louise Juvêncio Paes de Andrade (2019)	NH	Brazil	cross-sectional	321	242	79	NA	≥60	PFP	80.1
Tsuyoshi OKAMURA (2021)	NH	Japan	Cohort study	1736	NA	NA	NA	≥65	KCL	10.0-16.0
Aulia Rizka (2021)	NH	Indonesia	cross-sectional	214	143	71	68.0±4.3	≥60	PFP	46.5
Bader A. Alqahtani (2021)	Community	Saudi Arabia	cross-sectional	486	317	169	NA	≥60	PFP	21.4
Xingfeng Yu (2022)	Community	China	cross-sectional	2647	1087	1560	68.6±6.1	≥60	CFAI	70.6
Ah Ram Jang (2021)	Community	Korea	Cohort study	1428	689	739	77.9±3.8	≥70	FRAIL scale	14.1
Nur Sakinah Ahmad (2018)	Community	Malaysia	Cohort study	2413	887	1437	NA	≥60	PFP	9.2-9.8
Agnieszka Batko-Szwaczka (2020)	Community	Poland	cross-sectional	160	89	71	68.0±4.2	≥60	PFP	2.5
Takehiko Doi (2018)	Community	Japan	Cohort Study	4676	2316	2360	NA	≥65	PFP	7.6
Graziamaria corbi (2019)	Community	Italy	Cohort Study	1284	552	732	74.2±6.4	≥65	FSS	16.3
Zhou J (2023)	Community	China	Cohort Study	7471	3310	4161	89.4±6.6	≥80	FI	34.0
Ameneh Sobhani (2022)	Community	Iran	cross-sectional	1529	740	789	70.6±8.2	≥60	PFP	9.55
Lixia Ge (2022)	Community	Singapore	Cohort Study	606	257	349	NA	≥60	Clinical Frailty Scale	10.7
Siying Li (2022)	Community	China	Cohort Study	14314	6300	8014	NA	≥60	FI	27.2
Therri Usher (2021)	Community	The United States	Cohort Study	8245	3200	5045	74.7±0.3	≥65	PFP	81.3
Marcos Kaic Lopes Alves (2020)	Community	Brazil	cross-sectional	580	185	395	NA	≥60	PFP	13.1
L.L. Peters (2015)	Community	Netherlands	Cohort Study	12,706	2667	3045	NA	≥60	GFI	9.0
Jennifer Yee-man Tang (2021)	Community	China	cross-sectional	345	NA	NA	NA	≥60	FRAIL scale	41.0
Stefano Poli (2016)	Community	Italy	cross-sectional	542	259	283	79.6±5.1	≥65	FRAIL scale	15.1
O.H. DEL BRUTTO (2020)	Community	Ecuadorian	cross-sectional	324	138	186	70.5±8.0	≥60	EFS	21.0
Junyao Fan (2021)	Community	China	cross-sectional	454	192	262	69.3±6.7	≥60	TFI	40.7
Y. HENCHOZ (2016)	Community	Switzerland	Cohort Study	927	368	559	NA	≥65	PFP	3.3
Takaaki Ikeda (2019)	Community	Japan	Cohort Study	64496	19 092	19 605	NA	≥65	KCL	24.7
Hye-Young Jang (2021)	Community	Korea	cross-sectional	2340	455	1885	75.7±6.7	≥65	FRAIL scale	10.8
Heeeun Jung (2020)	Community	Korea	Cohort Study	2907	1383	1524	NA	≥70	PFP	45.2
Yu Kume (2021)	Community	Japan	Cohort Study	313	98	215	73.7±5.4	≥65	FI	3.8
Minhui Liu (2021)	Community	The United States	Cohort Study	7609	3171	4438	NA	≥65	PFP	14.0
Joaquim Oyon (2021)	Community	Spain	cross-sectional	338	61	277	77.2±5.0	≥70	PFP	36.1
Gulsah Ozsoy (2021)	Community	Turkey	cross-sectional	166	54	112	72.9±6.4	≥60	TFI, PFP	60.0-72.5
Siti Setiati (2021)	Community	Indonesia	Cohort Study	908	438	470	NA	≥60	FRAIL scale	18.4
Huai-yu Wang (2021)	Community	China	Cohort Study	3327	1684	1643	81.2±10.3	≥60	PFP	26.8
Liu Yang (2018)	Community	China	cross-sectional	306	169	137	NA	≥60	EFS	14.1
Karla Moreno-Tamayo (2021)	Community	Mexico	cross-sectional	591	NA	NA	76.3±3.3	≥70	PFP	10.7

Abbreviations: PFP, physical Fried's phenotype; KCL, Kihon Checklist; FSS, Frailty Staging System; FI, frailty index; DAI, Deficit Accumulation Index; GFI, Groningen Frailty indicator; EFS, Edmonton Frailty Scale; TFI, Tilburg Frailty Indicator; NH, nursing home; CFAI, Comprehensive Frailty Assessment Instrument.

Table S2. The estimated total uses and features of frailty instruments in the literature

Instrument	Nature	Features	Settings	No. of studies
PFP	uni-dimensional	unintentional weight loss, self-reported exhaustion, muscle weakness, slow walking speed and low physical activity	NH<br>Community	7<br>13
FRAIL scale	uni-dimensional	fatigue, resistance, ambulation, illnesses, loss of weight	Community	5
Clinical frailty scale	multidimensional	physical health, functional independence, social interaction, mental health, medical treatment	Community	1
KCL	multidimensional	physiological function, cognitive function, psychological well-being, social activities	NH<br>Community	1<br>1
FSS	multidimensional	disability, mobility, cognitive function, visual function, hearing function, urinary continence and social support	Community	1
FI/DAI	multidimensional	symptoms, illnesses, physical, and cognitive impairments, disabilities, psychosocial risk factors, and laboratory abnormalities	Community	4
GFI	multidimensional	physiological function, cognitive function, psychological well-being, social activities, activities of daily living (ADLs), physical activity level	Community	1
EFS	multidimensional	cognitive function, general health status, functional independence, social support, medication use, nutritional status, mood	Community	2
TFI	multidimensional	Part A comprises 10 questions on frailty determinants (e.g., age, gender, marital status, education level, and way of life);<br>Part B comprises 15 frailty elements arranged according to three different aspects.	Community	2
CFAI	multidimensional	physical, psychological, social and environmental	Community	1

Abbreviations: PFP, physical Fried's phenotype; KCL, Kihon Checklist; FSS, Frailty Staging System; FI, frailty index; DAI, Deficit Accumulation Index; GFI, Groningen Frailty indicator; EFS, Edmonton Frailty Scale; TFI, Tilburg Frailty Indicator; NH, nursing home; CFAI, Comprehensive Frailty Assessment Instrument.

Note：In cases where the same article utilizes different tools, a duplicate calculation is performed to address this scenario.
