# Supplementary material for: Factors Associated with Frailty in Older Adults in Community and Nursing Home Settings: A Systematic Review with a Meta-Analysis
Source: J Clin Med. 2024 Apr 19;13(8):2382. doi: 10.3390/jcm13082382 (PMC11050860; doi:10.3390/jcm13082382)
Supplement: Supplementary file 1 [file jcm-13-02382-s001.zip › Supplemental File S3 quailty assessment.pdf]

Figure S5 Risk of bias graph of the included cross-sectional studies

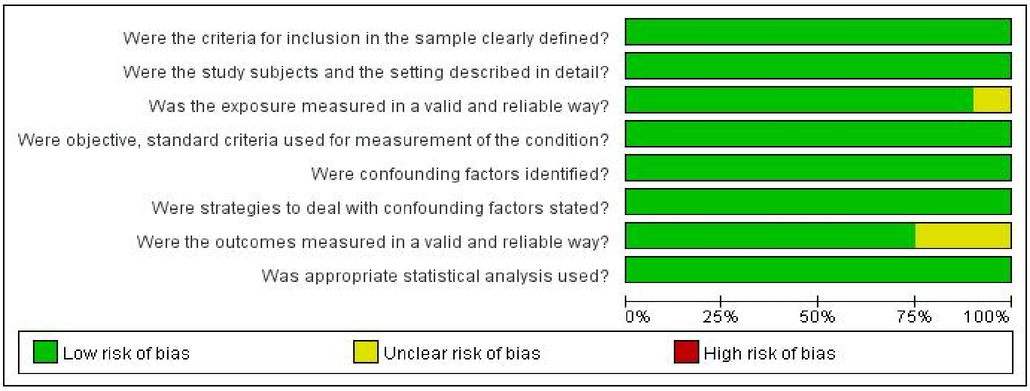

Figure S6 Risk of bias summary of the included cross-sectional studies

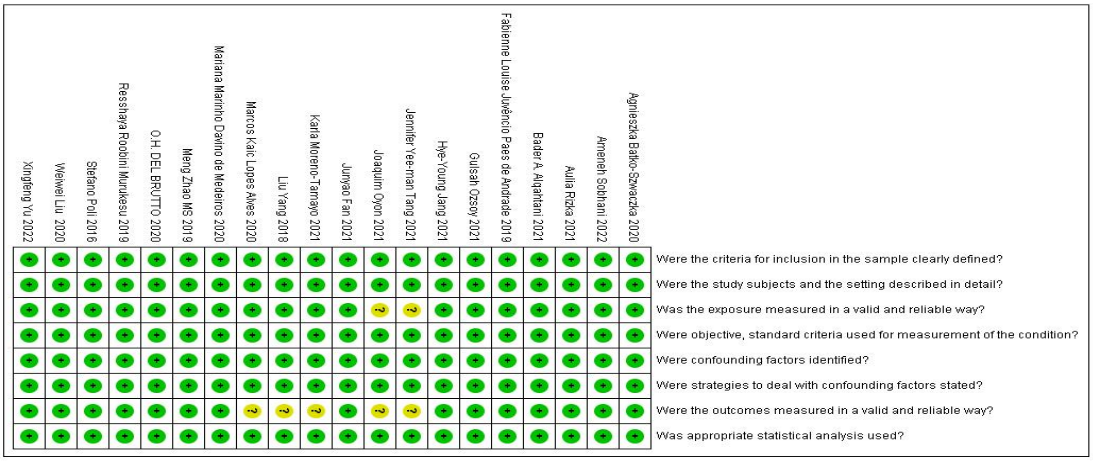

Figure S7 Risk of bias graph of the included cohort studies

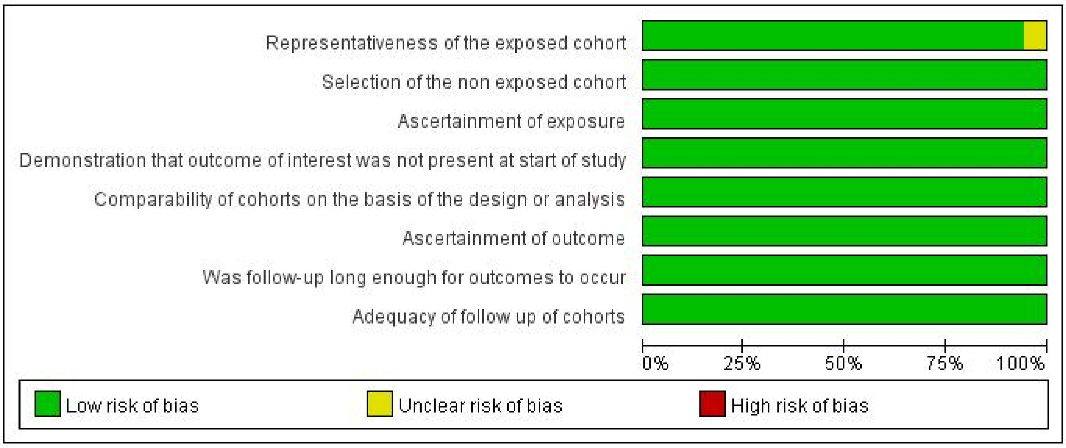

Figure S8 Risk of bias summary of the included cohort studies

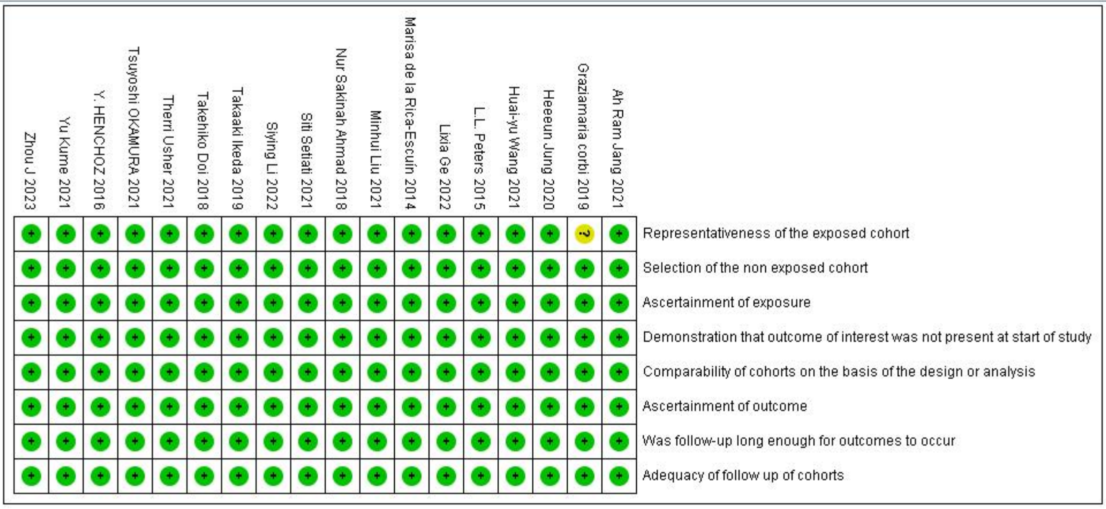
Zhou J 2023
Yu Kurne 2021
Y. HENCHOZ 2016
Tsuyoshi OKAMURA 2021
Therri Usher 2021
Takehiko Doi 2018
Takaaki Ikeda 2019
Siying Li 2022
Siti Setiati 2021
Nur Sakinah Ahmad 2018
Minhui Liu 2021
Marisa de la Rica-Escuín 2014
Lixia Ge 2022
L.L. Peters 2015
Huai-yu Wang 2021
Heeeun Jung 2020
Graziamaria corbi 2019
Ah Ram Jang 2021
Representativeness of the exposed cohort
Selection of the non exposed cohort
Ascertainment of exposure
Demonstration that outcome of interest was not present at start of study
Comparability of cohorts on the basis of the design or analysis
Ascertainment of outcome
Was follow-up long enough for outcomes to occur
Adequacy of follow up of cohorts
