# Supplementary material for: Factors Associated with Frailty in Older Adults in Community and Nursing Home Settings: A Systematic Review with a Meta-Analysis
Source: J Clin Med. 2024 Apr 19;13(8):2382. doi: 10.3390/jcm13082382 (PMC11050860; doi:10.3390/jcm13082382)
Supplement: Supplementary file 1 [file jcm-13-02382-s001.zip › Supplemental File S4 forest plots and bias assessment.pdf]

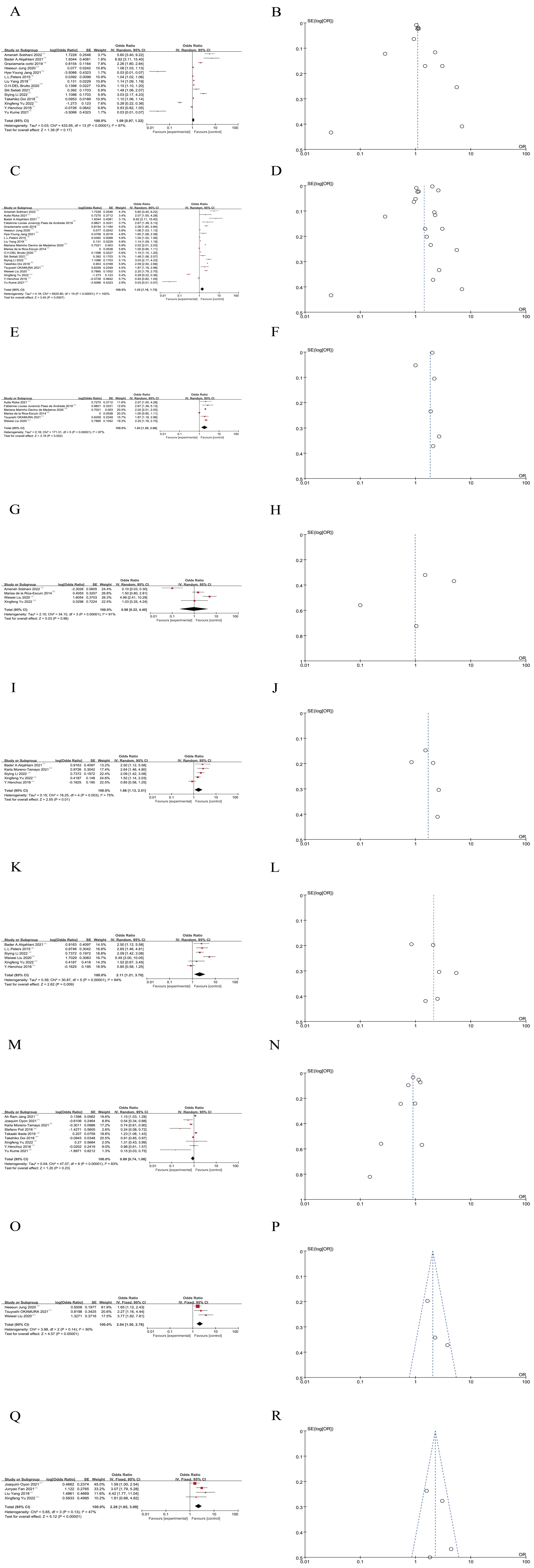
A
B
C
D
E
F
G
H
I
J
K
L
M
N
O
P
Q
R

Supplementary Figure S1. Meta-analysis of sociodemographic factors of frailty for [A,B] age(community), [C,D] age(community and nursing home), [E,F] age (nursing home), [G,H] female (comunity and nursing home), [I,J] living alone (community), [K,L] living alone (community and nursing home), [M,N] low education (community), [O,P] poo rself-reported health (community and nursing home), and [Q,R] Unmarried/divorced/widowed (community). Forest pl otis represented by mean with 95% confidence interval.

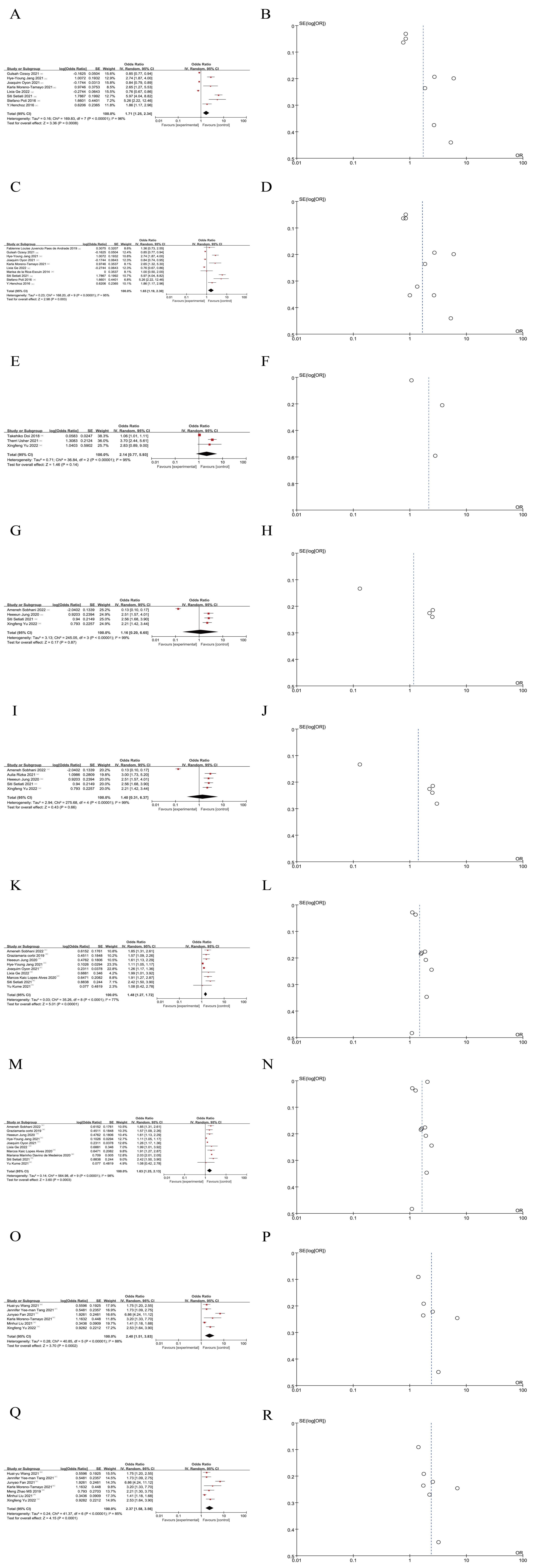

A
Study or Subgroup log[Odds Ratio] SE Weight Odds Ratio IV, Random, 95% CI
Gulsah Ozsoy 2021 -0.1625 0.0504 15.6% 0.85 [0.77, 0.94]
Hye-Young Jang 2021 1.0072 0.1932 12.9% 2.74 [1.87, 4.00]
Joaquim Oyon 2021 -0.1744 0.0313 15.8% 0.84 [0.79, 0.89]
Karla Moreno-Tamayo 2021 0.9746 0.3753 8.5% 2.65 [1.27, 5.53]
Lixia Ge 2022 -0.2744 0.0643 15.5% 0.76 [0.67, 0.86]
Siti Setiati 2021 1.7867 0.1992 12.7% 5.97 [4.04, 8.82]
Stefano Poli 2016 1.6601 0.4401 7.2% 5.26 [2.22, 12.46]
Y.Henchoz 2016 0.6206 0.2365 11.8% 1.86 [1.17, 2.96]
Total (95% CI) 100.0% 1.71 [1.25, 2.34]
Heterogeneity: Tau² = 0.16; Chi² = 169.83, df = 7 (P < 0.00001); I² = 96%
Test for overall effect: Z = 3.36 (P = 0.0008)
Favours [experimental] Favours [control]
B
SE(log[OR])
OR
C
Study or Subgroup log[Odds Ratio] SE Weight Odds Ratio IV, Random, 95% CI
Fabienne Louise Juvencio Paes de Andrade 2019 0.3075 0.3207 8.6% 1.36 [0.73, 2.55]
Gulsah Ozsoy 2021 -0.1625 0.0504 12.4% 0.85 [0.77, 0.94]
Hye-Young Jang 2021 1.0072 0.1932 10.8% 2.74 [1.87, 4.00]
Joaquim Oyon 2021 -0.1744 0.0313 12.5% 0.84 [0.79, 0.89]
Karla Moreno-Tamayo 2021 0.9746 0.3537 8.1% 2.65 [1.32, 5.30]
Lixia Ge 2022 -0.2744 0.0643 12.3% 0.76 [0.67, 0.86]
Marisa de la Rica-Escuín 2014 0 0.3537 8.1% 1.00 [0.50, 2.00]
Siti Setiati 2021 1.7867 0.1992 10.7% 5.97 [4.04, 8.82]
Stefano Poli 2016 1.6601 0.4401 6.8% 5.26 [2.22, 12.46]
Y.Henchoz 2016 0.6206 0.2365 10.1% 1.86 [1.17, 2.96]
Total (95% CI) 100.0% 1.65 [1.19, 2.30]
Heterogeneity: Tau² = 0.23; Chi² = 188.20, df = 9 (P < 0.00001); I² = 95%
Test for overall effect: Z = 2.98 (P = 0.003)
Favours [experimental] Favours [control]
D
SE(log[OR])
OR
E
Study or Subgroup log[Odds Ratio] SE Weight Odds Ratio IV, Random, 95% CI
Takehiko Doi 2018 0.0583 0.0247 38.3% 1.06 [1.01, 1.11]
Therri Usher 2021 1.3083 0.2124 36.0% 3.70 [2.44, 5.61]
Xingfeng Yu 2022 1.0403 0.5902 25.7% 2.83 [0.89, 9.00]
Total (95% CI) 100.0% 2.14 [0.77, 5.93]
Heterogeneity: Tau² = 0.71; Chi² = 36.84, df = 2 (P < 0.00001); I² = 95%
Test for overall effect: Z = 1.46 (P = 0.14)
Favours [experimental] Favours [control]
F
SE(log[OR])
OR
G
Study or Subgroup log[Odds Ratio] SE Weight Odds Ratio IV, Random, 95% CI
Ameneh Sobhani 2022 -2.0402 0.1339 25.2% 0.13 [0.10, 0.17]
Heeeun Jung 2020 0.9203 0.2394 24.9% 2.51 [1.57, 4.01]
Siti Setiati 2021 0.94 0.2149 25.0% 2.56 [1.68, 3.90]
Xingfeng Yu 2022 0.793 0.2257 24.9% 2.21 [1.42, 3.44]
Total (95% CI) 100.0% 1.16 [0.20, 6.65]
Heterogeneity: Tau² = 3.13; Chi² = 245.05, df = 3 (P < 0.00001); I² = 99%
Test for overall effect: Z = 0.17 (P = 0.87)
Favours [experimental] Favours [control]
H
SE(log[OR])
OR
I
Study or Subgroup log[Odds Ratio] SE Weight Odds Ratio IV, Random, 95% CI
Ameneh Sobhani 2022 -2.0402 0.1339 20.2% 0.13 [0.10, 0.17]
Aulia Rizka 2021 1.0986 0.2809 19.8% 3.00 [1.73, 5.20]
Heeeun Jung 2020 0.9203 0.2394 20.0% 2.51 [1.57, 4.01]
Siti Setiati 2021 0.94 0.2149 20.0% 2.56 [1.68, 3.90]
Xingfeng Yu 2022 0.793 0.2257 20.0% 2.21 [1.42, 3.44]
Total (95% CI) 100.0% 1.40 [0.31, 6.37]
Heterogeneity: Tau² = 2.94; Chi² = 275.68, df = 4 (P < 0.00001); I² = 99%
Test for overall effect: Z = 0.43 (P = 0.66)
Favours [experimental] Favours [control]
J
SE(log[OR])
OR
K
Study or Subgroup log[Odds Ratio] SE Weight Odds Ratio IV, Random, 95% CI
Ameneh Sobhani 2022 0.6152 0.1761 10.8% 1.85 [1.31, 2.61]
Graziamaria corbi 2019 0.4511 0.1848 10.2% 1.57 [1.09, 2.26]
Heeeun Jung 2020 0.4762 0.1806 10.5% 1.61 [1.13, 2.29]
Hye-Young Jang 2021 0.1026 0.0294 23.3% 1.11 [1.05, 1.17]
Joaquim Oyon 2021 0.2311 0.0378 22.8% 1.26 [1.17, 1.36]
Lixia Ge 2022 0.6881 0.346 4.2% 1.99 [1.01, 3.92]
Marcos Kaio Lopes Alves 2020 0.6471 0.2082 8.8% 1.91 [1.27, 2.87]
Siti Setiati 2021 0.8838 0.244 7.1% 2.42 [1.50, 3.90]
Yu Kume 2021 0.077 0.4819 2.3% 1.08 [0.42, 2.78]
Total (95% CI) 100.0% 1.48 [1.27, 1.72]
Heterogeneity: Tau² = 0.03; Chi² = 35.26, df = 8 (P < 0.0001); I² = 77%
Test for overall effect: Z = 5.01 (P < 0.00001)
Favours [experimental] Favours [control]
L
SE(log[OR])
OR
M
Study or Subgroup log[Odds Ratio] SE Weight Odds Ratio IV, Random, 95% CI
Ameneh Sobhani 2022 0.6152 0.1761 10.5% 1.85 [1.31, 2.61]
Graziamaria corbi 2019 0.4511 0.1848 10.3% 1.57 [1.09, 2.26]
Heeeun Jung 2020 0.4762 0.1806 10.4% 1.61 [1.13, 2.29]
Hye-Young Jang 2021 0.1026 0.0294 12.7% 1.11 [1.05, 1.17]
Joaquim Oyon 2021 0.2311 0.0378 12.6% 1.26 [1.17, 1.36]
Lixia Ge 2022 0.6881 0.346 7.0% 1.99 [1.01, 3.92]
Marcos Kaio Lopes Alves 2020 0.6471 0.2082 9.8% 1.91 [1.27, 2.87]
Mariana Marinho Davino de Medeiros 2020 0.709 0.005 12.8% 2.03 [2.01, 2.05]
Siti Setiati 2021 0.8838 0.244 9.0% 2.42 [1.50, 3.90]
Yu Kume 2021 0.077 0.4819 4.9% 1.08 [0.42, 2.78]
Total (95% CI) 100.0% 1.63 [1.25, 2.13]
Heterogeneity: Tau² = 0.14; Chi² = 564.98, df = 9 (P < 0.00001); I² = 98%
Test for overall effect: Z = 3.60 (P = 0.0003)
Favours [experimental] Favours [control]
N
SE(log[OR])
OR
O
Study or Subgroup log[Odds Ratio] SE Weight Odds Ratio IV, Random, 95% CI
Huai-yu Wang 2021 0.5596 0.1925 17.9% 1.75 [1.20, 2.55]
Jennifer Yee-man Tang 2021 0.5481 0.2357 16.9% 1.73 [1.09, 2.75]
Junyao Fan 2021 1.9261 0.2461 16.6% 6.86 [4.24, 11.12]
Karla Moreno-Tamayo 2021 1.1632 0.448 11.8% 3.20 [1.33, 7.70]
Minhui Liu 2021 0.3436 0.0909 19.7% 1.41 [1.18, 1.68]
Xingfeng Yu 2022 0.9282 0.2212 17.2% 2.53 [1.64, 3.90]
Total (95% CI) 100.0% 2.40 [1.51, 3.83]
Heterogeneity: Tau² = 0.28; Chi² = 40.85, df = 5 (P < 0.00001); I² = 88%
Test for overall effect: Z = 3.70 (P = 0.0002)
Favours [experimental] Favours [control]
P
SE(log[OR])
OR
Q
Study or Subgroup log[Odds Ratio] SE Weight Odds Ratio IV, Random, 95% CI
Huai-yu Wang 2021 0.5596 0.1925 15.5% 1.75 [1.20, 2.55]
Jennifer Yee-man Tang 2021 0.5481 0.2357 14.5% 1.73 [1.09, 2.75]
Junyao Fan 2021 1.9261 0.2461 14.3% 6.86 [4.24, 11.12]
Karla Moreno-Tamayo 2021 1.1632 0.448 9.8% 3.20 [1.33, 7.70]
Meng Zhao MS 2019 0.793 0.2703 13.7% 2.21 [1.30, 3.75]
Minhui Liu 2021 0.3436 0.0909 17.3% 1.41 [1.18, 1.68]
Xingfeng Yu 2022 0.9282 0.2212 14.9% 2.53 [1.64, 3.90]
Total (95% CI) 100.0% 2.37 [1.58, 3.56]
Heterogeneity: Tau² = 0.24; Chi² = 41.37, df = 6 (P < 0.0001); I² = 85%
Test for overall effect: Z = 4.15 (P < 0.0001)
Favours [experimental] Favours [control]
R
SE(log[OR])
OR

Supplementary Figure S2. Meta-analysis of physiological factors of frailty for [A,B] low ADL (community), [C,D] low ADL(community and nursing home), [E,F] low BMI (community), [G,H] malnutrition (community), [I,J] malnutrition (community and nursing home), [K,L] poly pharmacy (community), [M,N] poly pharmacy (community and nursin ghome), [O,P] poor sleep (community), and [Q,R] poor sleep (community and nursing home). Forest plot is represent edby mean with 95% confidence interval.

A

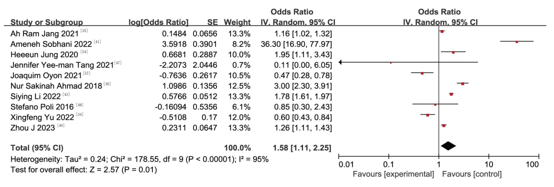

B

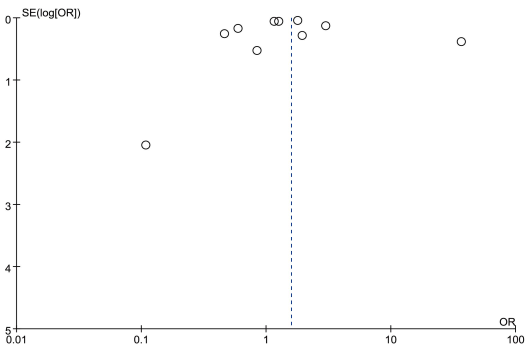

C

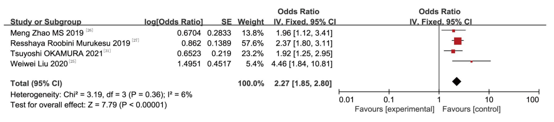

D

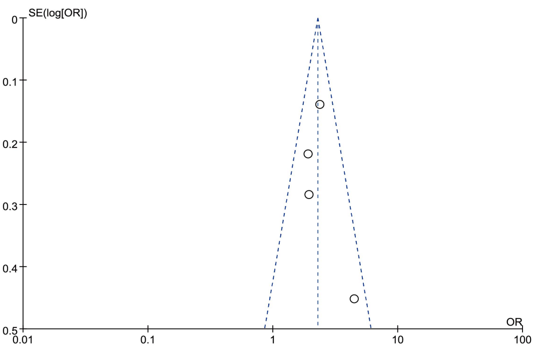

E

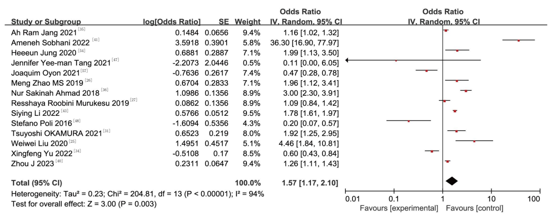

F

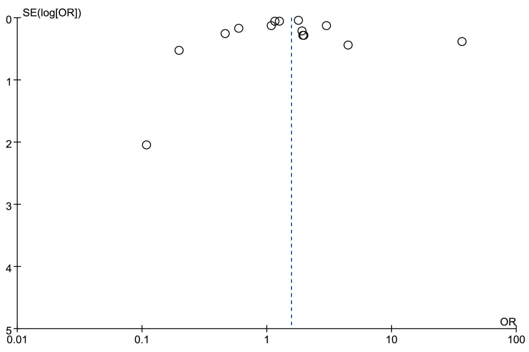

Supplementary Figure S3. Meta-analysis of behavior factors of frailty for [A,B] physical inactivity (community), [C,D] physical inactivity(nursing home), and [E,F] physical inactivity (community and nursing home). Forest plot is represented by mean with 95% confidence interval.

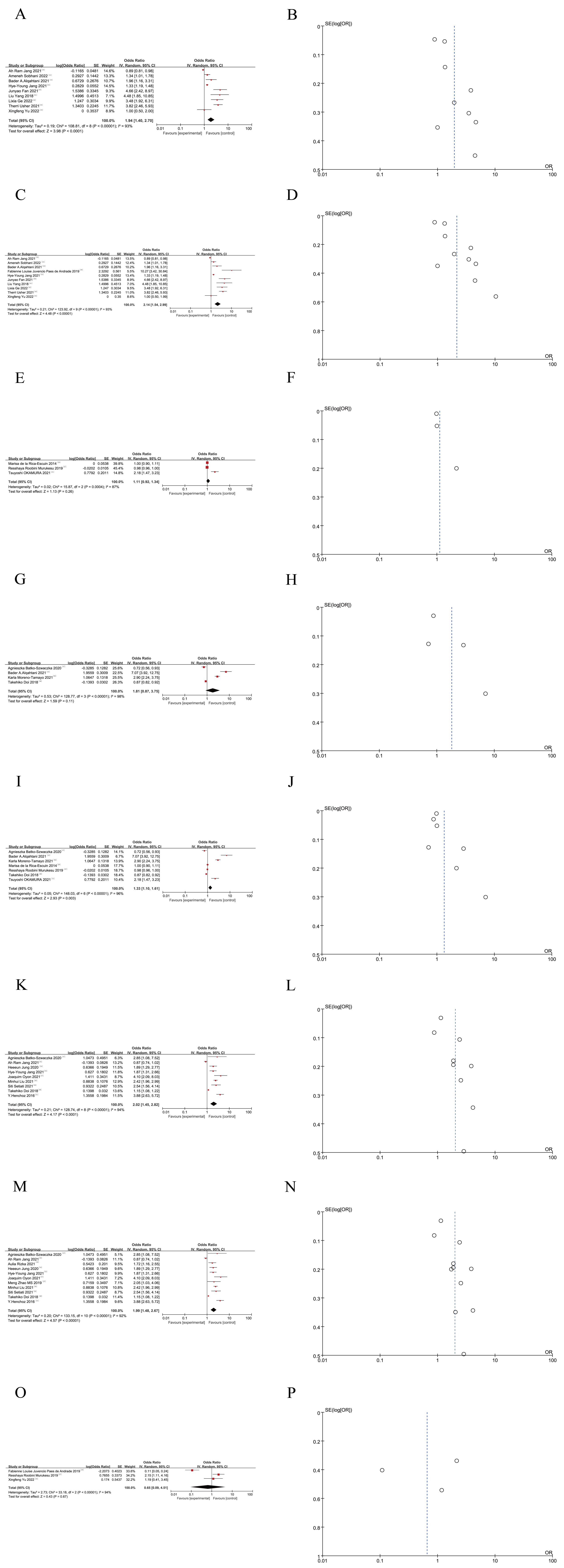

Supplementary Figure S4. Meta-analysis of disease factors of frailty for [A,B] chronic conditions (community), [C,D] chronic conditions(community and nursing home), [E,F] cognitive impairment (nursing home), [G,H] cognitive

impairment (community), [I,J] cognitive impairment (community and nursing home), [K,L] depression (communit y),[M,N] depression (community and nursing home), [O,P] hypertension (community and nursing home). Forest pl ot isrepresented by mean with 95% confidence interval.
